# Compliance with Cardiovascular Prevention Guidelines in Type 2 Diabetes Individuals in a Middle-Income Region: A Cross-Sectional Analysis

**DOI:** 10.3390/diagnostics12040814

**Published:** 2022-03-26

**Authors:** Joaquim Barreto, Beatriz Luchiari, Vaneza L. W. Wolf, Isabella Bonilha, Ticiane G. Bovi, Barbara S. Assato, Ikaro Breder, Sheila T. Kimura-Medorima, Daniel B. Munhoz, Thiago Quinaglia, Otavio R. Coelho-Filho, Luiz Sergio F. Carvalho, Wilson Nadruz, Andrei C. Sposito

**Affiliations:** 1 Atherosclerosis and Vascular Biology Laboratory (Aterolab), Division of Cardiology, State University of Campinas (Unicamp), Sao Paulo 13083-894, Brazil; joaquimbarretoantunes@gmail.com (J.B.); beatriz.m.luchiari@gmail.com (B.L.); vanessawolf@hotmail.com (V.L.W.W.); isaoliveira.ib@gmail.com (I.B.); ticianebovi@hotmail.com (T.G.B.); barbarasanae@gmail.com (B.S.A.); ikarobreder@gmail.com (I.B.); sheilatk@gmail.com (S.T.K.-M.); dbmunhoz@gmail.com (D.B.M.); tquinaglia@yahoo.com.br (T.Q.); 2 Division of Cardiology, Department of Internal Medicine, Unicamp, Sao Paulo 13083-970, Brazil; tavicocoelho@gmail.com (O.R.C.-F.); wilnj@unicamp.br (W.N.); 3 Clarity Healthcare Intelligence, Jundiaí 13214-658, Brazil; luizsergiofc@gmail.com

**Keywords:** diabetes, goal attainment, cardiovascular risk

## Abstract

Stricter control of risk factors has been pursued as a compelling strategy to mitigate cardiovascular events (CVE) in type 2 diabetes (T2D) individuals. However, the achievement rate of the recommended goals has remained low in clinical practice. This study investigated the 2019 ESC guideline recommendation attainment among T2D individuals enrolled in a national cohort held in Brazil. Data from 1030 individuals (mean age: 58 years old; 54% male; mean T2D duration: 9.7 years) were analyzed. The control rates were 30.6% for SBP, 18.8% for LDL-C, and 41% for A1c, and only 3.2% of the study participants met all three targets. Statins and high-intensity lipid-lowering therapy prescription rates were 45% and 8.2%, respectively. Longer T2D duration and those at higher CV risk were less likely to be controlled. Longer diabetes duration and higher CV risk were inversely related to the chance of achieving the recommended targets. Treatment escalation using conventional therapies would be sufficient to gain optimal control in most of the study sample. In conclusion, a minimal proportion of T2D individuals comply with guidelines-oriented CV prevention targets. Given the significant burden of the disease, and the substantial effect size predicted for these therapies, bridging this gap between guidelines and clinical practice should be considered an urgent call to public health managers.

## 1. Introduction

The global burden of type 2 diabetes (T2D) has imposed an unprecedented challenge on public health [1] The International Federation of Diabetes estimates a global prevalence of 537 million individuals with T2D, projected to reach 783 million individuals before 2045 [1]. This scenario suggests 6.3 million deaths and USD 1.42 trillion in healthcare expenditure every year [2]. In this population, cardiovascular disease (CVD) remains the leading cause of death and morbidity, accounting for half of the deaths and direct costs of T2D treatment [3]. Due to these figures, strategies to mitigate mortality and morbidity caused by T2D have become imperative [1].

Glycemic control has been pursued as a tempting target to attenuate growing cardiovascular risk among T2D individuals [4]. This strategy is grounded by prior studies demonstrating that intensive glucose control, defined as A1c <7%, attenuates the 10-year incidence of myocardial infarction by 42% and all-cause mortality by 36% compared to conventional glucose control [4]. This approach has mitigated but has not nullified the growing incidence of CVE in T2D compared to non-T2D subjects [5].

Hypertension and dyslipidemia frequently overlap in T2D, significantly impacting CV mortality [6,7]. Data from experimental studies shows that insulin resistance, a prominent feature of T2D, accentuates the progression of endothelial dysfunction, hyperactivates the renin–angiotensin–aldosterone system, and prompts autonomic dysfunction, all leading to SBP increase [8,9]. As a result, hypertension is twice more prevalent in T2D, affecting up to 80% of T2D subjects, and hypertensive T2D individuals display a 35% increased risk of CVE compared to normotensive counterparts [9,10]. This risk may, however, be attenuated by BP control, as each 10 mm Hg decrease in SBP yields an estimated 17% reduction in the incidence of CVE [11].

The association of T2D and dyslipidemia is a common feature that suggests increased CV risk [12]. In this matter, T2D through insulin resistance favors the bioavailability of free fatty acids involved in generating ApoB-containing lipoprotein. Moreover, T2D subjects display higher levels of oxidized low-density lipoprotein (oxLDL), which binds to scavenger receptors favoring accelerated atherosclerosis [13,14]. These events are attenuated by the reduction of LDL-C levels, which is chiefly met by the intensification of LLT, either by switching statin regimes or adding non-statin LLT agents, such as ezetimibe and PCSK9 inhibitors, that yield incremental drops in the incidence of CVE as high as 35% [15,16,17]. In fact, an estimated 21% relative risk reduction in the incidence of CVE is expected per 1 mmol/L drop in LDL-C levels [18].

Although the development of new therapies is essential to abrogate residual risk, the addition of new approaches presupposes the proper use of existing evidence. However, observational studies have consistently demonstrated that T2D subjects seldomly comply with current guidance. In this matter, prior reports primarily conducted in high-income countries found attainment rates of 43% for A1c, 29% for SBP, and 49% for LDL-C targets, as well as the suboptimal prescription of therapies directed to the achievement of these goals [19]. Presumably, this scenario is aggravated in low- and middle-income countries, which together account for 80% of the population living with T2D, as these countries display narrowed access to medical care, and lower level of education and therapy adherence, all of which are features of guideline non-compliance [20,21].

In this context, we designed this study to assess the rate of adherence to CVD prevention goals in a cohort of adults with T2D across a broad spectrum of socioeconomic status in Brazil. In this middle-income country, T2D prevalence has reached the concerning number of 16 million adults, accounting for 4% of the global prevalence of T2D [1]. Moreover, recently, it has been demonstrated that Brazilian T2D individuals are susceptible to a 28% increased risk of CVD compared to T2D individuals from developed countries [22]. We sought to assess how reasonably Brazilian T2D individuals comply with guideline-recommended targets and estimate the possibility of controlling these factors by escalating current therapies.

## 2. Materials and Methods

### 2.1. Study Population

The study was designed in an observational, cross-sectional manner, conducted with participants from the Brazilian Diabetes Study (clinicaltrials.gov: NCT04949152), an ongoing prospective cohort headed by the Brazilian Heart Study Group at the Clinical Research Center of the State University of Campinas (UNICAMP), Brazil. The study population included individuals with T2D aged 30 or more. The volunteers were recruited between 2016 and 2021 through radio, newspaper, television, and social media advertisements. A total of 1030 participants were enrolled in this analysis. The local ethics committee approved the study (CAAE: 41618915.1.0000.5404), and all patients signed an informed consent form. Study data were collected and managed using REDCap electronic data capture tools.

All participants were enrolled in interviews at the clinical research center with investigators to collect demographical data, medical history, and physical examination. Demographical data included age, self-reported race, family income, years of study, and marital status. Medical history comprised register of CVD (coronary artery disease, transient ischemic attack, prior stroke, or peripheral artery disease), smoking status, hypertension (previously known diagnosis or in use of any antihypertensive drugs), dyslipidemia (previous diagnosis or use of LLT), and current name and dosing of medications in use. On this occasion, patients underwent SBP measurement [23], ophthalmological examination [24], and peripheral blood and urine sample collection for biochemical analysis. The study design has been detailed elsewhere [25].

### 2.2. Definition of Cardiovascular Risk and Goals

Participants were grouped as very high (VHR), high (HR), and moderate (MR) CV risk according to the 2019 ESC/EAS guidelines [26]. Briefly, VHR was considered given any of the following: prior CVD, target-organ damage (proteinuria, renal impairment defined as estimated glomerular filtration rate (eGFR) <30 mL/min/1.73 m [2], or retinopathy), or three or more major risk factors (age >50 years old, hypertension, dyslipidemia, smoking, and obesity). HR was defined as more than 10 years of T2D in addition to one or more risk factors. MR was considered if <10 years of T2D, age <50 years old, and no predefined risk factor [26].

Risk factor control goals were based on the 2019 ESC/EAS guidelines [26]. SBP goal was <130 mm Hg for most individuals, and <140 mm Hg when ≥65 years old. Targeted LDL-C were <55 mg/dL, <70 mg/dL and <100 mg/dL for VHR, HR and MR, respectively. A1c targets were <7% (if <65 years old), or <8% (if ≥65 years old).

As cohort recruitment began before the release of the 2019 ESC guidelines, we also disclosed the achievement rate of the 2013 ESC/EAS guideline recommendations. These guideline-recommended SBP and HbA1c levels were <140 mm Hg and <7%, respectively, and LDL-C <70 mg/dL for VHR and <100 mg/dL for HR subjects. No specific LDL-C goal was established for MR individuals.

### 2.3. Estimate Change in Attainment Rate According to Intervention

LLT drugs comprised statins and ezetimibe. Statins were grouped according to the expected mean relative reduction of LDL-C as low- to moderate-intensity statins (MIS; simvastatin 10–40 mg/day, atorvastatin 10–20 mg/day or rosuvastatin 5–10 mg/day) and high-intensity statins (HIS; atorvastatin 40–80 mg/day or rosuvastatin 20-4) as previously validated [27]. Ezetimibe was either as monotherapy (Ez) or combined to MIS (MIS + Ez) or HIS (HIS + Ez). Individuals who were not on statins or ezetimibe at baseline were classified as statin-naïve (NoS). For each LLT regimen, distance to the target (DDT) was calculated as the absolute difference between observed and targeted LDL-C. Based on these values, we estimated the % change in LDL-C control according to the expected reduction in LDL-C obtained by treatment intensification according to the 2019 ESC guideline estimates [26].

Antihypertensive therapy (AHT) was registered according to the number of classes used. Based on current treatment, we estimated the mean absolute reduction in SBP that would have been achieved by treatment intensification according to the *2018 ESC Guidelines on Arterial Hypertension* [28]. Most importantly, among AHD-naïve subjects, the expected SBP reduction achieved by monotherapy initiation, with either an ACEi or ARB, would be 27 mm Hg. In contrast, escalation of this regimen to dual (thiazide diuretic or CCB on top of ACEi or ARB) and triple therapy (ACEi or ARB, thiazide diuretic and CCB) would yield incremental reductions of 11 mm Hg and 10 mm Hg, respectively [29,30,31]. In individuals whose SBP remained above the threshold after triple therapy, spironolactone as the fourth drug would yield a mean decrease from baseline in SBP of 8.7 mm Hg [28,32].

Antidiabetic therapy (ADT) was registered according to pharmacological class and the number of drugs used. The anticipated A1c reduction obtained by treatment intensification was estimated for each regimen according to the 2021 ADA Guidelines [33]. In this analysis, in ADT-naïve subjects, monotherapy initiation would reduce A1c by 1.5%, while the addition of ADT drugs would yield additive reductions of 0.6% per added class [33,34,35,36]. The sequential addition of drugs considered metformin as monotherapy, Sglt2i, GLP1-A, and pioglitazone or DPP4 inhibitor for treatment escalation [33].

### 2.4. Statistical Analysis

Data are mean ± standard deviation for normally distributed data or median (interquartile range) for skewed data. Means were compared by Student’s T-test or one-way ANOVA for continuous variables and chi-square or Fisher’s Exact Test for categorical variables. Medians were compared by Kruskal–Wallis and Mann–Whitney test. Binary logistic regression was used to estimate the odds ratio with 95% confidence intervals. Adjusted OR were calculated using independent variables as covariates and assessed separately for each dependent variable according to the risk factor or combination of controlled factors of interest. A two-sided *p*-value of 0.05 was considered statistically significant. Statistical analyses were performed using IBM SPSS Statistics for Mac version 20.0 (Armonk, NY, USA: IBM Corp).

## 3. Results

**Figure 1:**
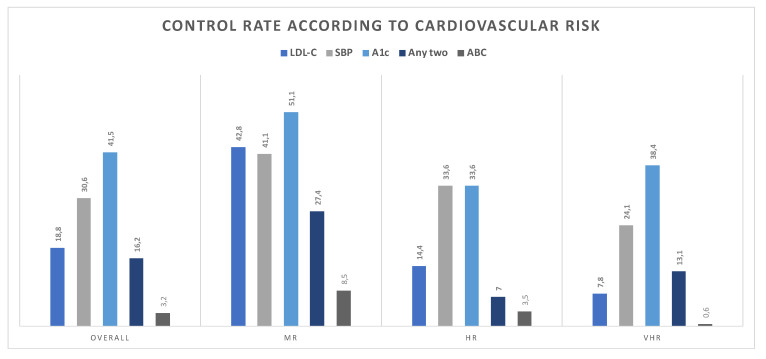
Control rate according to the risk factor and baseline cardiovascular risk. MR, moderate risk; HR, high risk; VHR, very high risk; T2D, type 2 diabetes; SBP, systolic blood pressure; A1c, glycated hemoglobin; ABC, HbA1c, blood pressure, and LDL-C targets combined.

This study included 1030 individuals whose baseline characteristics are summarized in Table 1 and presented in detail in the supplementary material (Table S1). In this population, the mean age was 58 years, T2D duration averaged 9.7 years, and 59% were male. The prevalence of obesity was 45%, 6.7% were smokers, and 17.4% had prior CVD. Hypertension, retinopathy, and proteinuria affected 81%, 21%, and 19.7% of the study sample, respectively. SBP averaged 141 mm Hg, whereas mean values of A1c and LDL-C were 7.9% and 107 mg/dL, respectively. LLT was prescribed to 45% [MIS (36.5%), HIS (5.9%), MIS-Ez (1.3%), HiS-Ez (1.0%), Ez (0.5%)] and 64.4% were on AHT. A total of 98.5% of enrollees used an oral ADT, 19.5% were on insulin therapy, and 16.6% took cardioprotective ADT. In this population, the attainment rate of recommended goals was 30.6% for SBP, 18.8% for LDL-C, and 41.2% for A1c. The percentage of individuals who reached none, one, two, and all three targets combined were 38%, 42%, 16%, and 3.2% (Figure 1).

Patients were then grouped according to their baseline CVR as MR (30.5%), HR (15%), and VHR (54%). Compared to MR and HR individuals, VHR individuals showed longer T2D duration, were older, less educated, and had lower family income (Table 1). Compared to MR, VHR individuals showed lower attainment of each of the evaluated variables [42.8% vs. 7.8% for LDL-C, 23% vs. 16.1% for SBP, and 51% vs. 38% for A1c; *p* < 0.050 for all]. Compliance with any two targets was 27.4% in MR, 7% in HR, and 13.1% among VHR individuals, and achievement of all three targets comprised 8.5%, 3.5%, and 0.6% of these patients.

**Figure 2:**
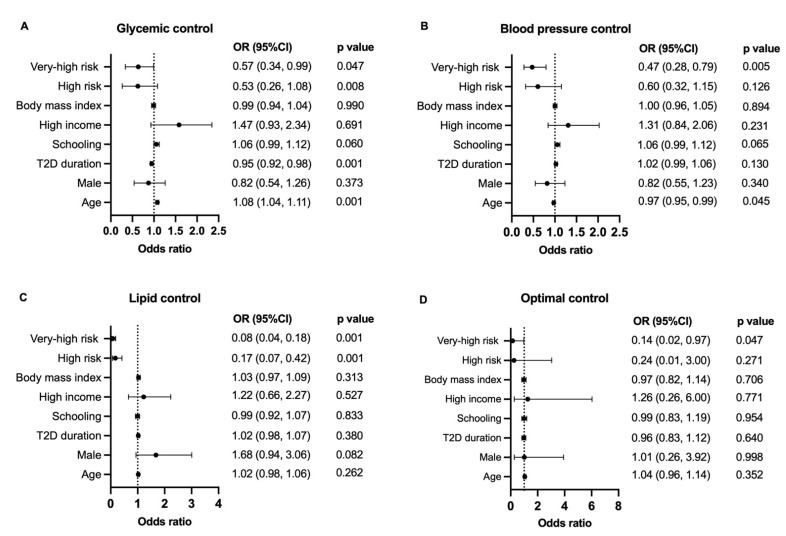
Forest plot presenting the result of adjusted logistic regression analysis for target achievement (**A**) A1c, (**B**) SBP, (**C**) LDL-C, (**D**) all three targets. Forest plot figures for logistic regression analysis. BMI, body mass index; CI, confidence intervals.

A binary logistic regression using each target as the dependent variable was used to assess the factors related to target achievement in this population. The results of this analysis are summarized in Figure 2, and absolute values are presented in the supplementary material (Table S2). Glycemic control was positively related to age and inversely associated with T2D duration and CVR. SBP target achievement rate was negatively influenced by age and CVR, whereas LDL-C goals were less often achieved by individuals at higher CV risk. Achievement of all three targets together was less common among VHR individuals than in MR subjects with an OR of 0.146 (95%CI: 0.02, 0.97; *p* = 0.047).

**Figure 3:**
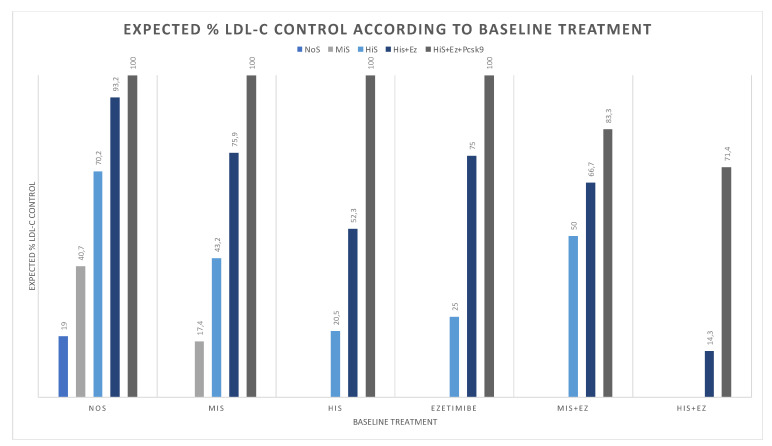
Baseline and expected relative change in LDL-C control rate according to current and escalated therapies. NOS, statin-naïve; MIS, moderate-intensity statin; HIS, high-intensity statin; MIS + Ez, MiS combined with ezetimibe; HiS + Ez, HIS combined with ezetimibe; HbA1c, glycated hemoglobin.

To ascertain the potential impact of LLT escalation on goal achievement rate, 677 patients were classified based on their current LLT therapy as NoS, MIS, HIS, MIS-Ez, HiS-Ez, and Ez. Among NoS (mean: 113 mg/dL, control: 19%), LDL-C control would significantly increase to 40% after MIS (*p* < 0.001), 70% after HiS (*p* < 0.001) and to 93% after HiS-Ez (*p* < 0.001). Among MIS (mean: 101 mg/dL, control: 17%), up-titration to HiS and HiS-Ez would yield raise control rates to 43% (*p* < 0.001) and 76% (*p* < 0.001), respectively. For all on statins or ezetimibe monotherapy, prescription of a Pcks9i add-on to HiS-Ez would be sufficient to achieve 100% of LDL-C control. Contrastingly, in those already on a combined therapy (MIS-Ez or HiS-Ez), Pcsk9i initiation would yield control rates between 71 and 83%. (*p* < 0.001 for both) (Figure 3).

**Figure 4:**
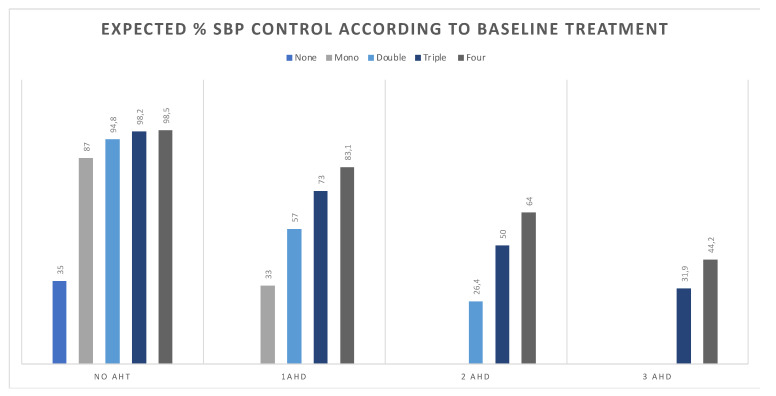
Baseline and expected relative change in SBP control according to current and escalated therapy. AHT, antihypertensive therapy; AHD, antihypertensive drugs; SBP, systolic blood pressure.

The estimated change from baseline in SBP control rate was ascertained in 903 enrollees. In this analysis, AHT-naïve (mean: 138 mm Hg, control: 35%) would benefit from initiation of AHT reaching SBP control rates as high as 98% after triple therapy (*p* < 0.001), and of 87% with monotherapy (*p* < 0.001). Similarly, among individuals initially on monotherapy (mean: 142 mm Hg; control: 33%), the control rate would consistently increase for each AHT added to the current regimen (57%, 73%, and 83%, for double, triple, and four AHT, respectively; *p* < 0.001). For individuals on double therapy (mean: 143 mm Hg, control: 26%), SBP control would increase from 26.4% to 50% with a triple (*p* = 0.004), and to 64% were a fourth drug combined (*p* = 0.005). In the subset of patients who were already on triple therapy, the addition of spironolactone would increase the control rate from 32% to 44% (*p* = 0.048) (Figure 4).

**Figure 5:**
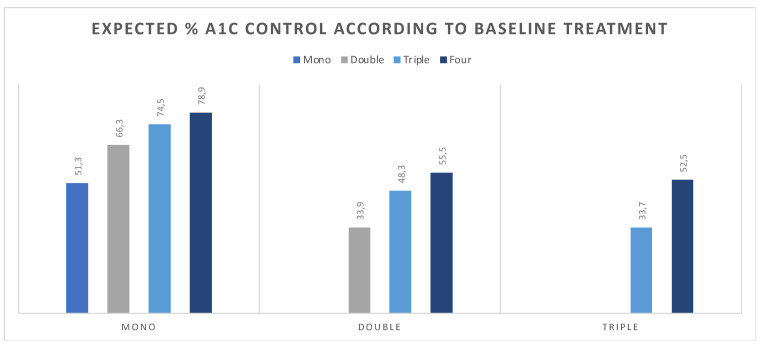
Baseline and expected % change in A1c control according to current therapy and estimated mean reduction in A1c with intervention.

The expected change in A1c control was assessed in 752 participants. The percentage of individuals with controlled A1c was 51% for monotherapy, 34% for dual therapy, 34% for triple, and 23% for insulin users. Among ADT monotherapy users, initiation of double, triple, and four drugs would increase A1c control to 66% (*p* = 0.031), 75% (*p* < 0.001), and 79% (*p* < 0.001), respectively. Similarly, escalating from double to triple therapy would yield a 48% control rate (*p* = 0.044), while four drugs would lead to a 56% (*p* = 0.020). Similarly, among individuals on triple therapy at baseline, adding a fourth drug would bear 53% A1c control (*p* = 0.006) (Figure 5).

Finally, the target achievement rate of the 2013 ESC guidelines was assessed. Overall, SBP and A1c targets were met by 51.5% and 37.1% of enrollees, respectively. The lipid control rate was 21.9%, ranging from 16.8% for VHR to 47.9% for HR subjects. The percentage of individuals meeting none, one, two, and all three targets were 35.6%, 42.3%, 17.2%, and 4.9%, respectively.

## 4. Discussion

To the best of our knowledge, this study is the first to evaluate the attainment of CV prevention guidelines and prescription patterns of T2D individuals in a developing middle-income country. Our findings shed light on a concerning reality: only 3.2% of T2D individuals, and 0.6% of VHR participants, had optimal risk factor control. Despite such figures, prescription patterns were inadequate for most patients, with goal nonachievers frequently on low-potency statins or monotherapy for A1c and SBP control. We estimated that using conventional therapies for treatment intensification would improve this scenario.

Randomized clinical trials have set lipid-lowering therapy as an imperative component of CV prevention in T2D [37]. In this matter, data from the CARDS study showed that the initiation of atorvastatin 10 mg/day in statin-naïve individuals reduced by 36% the 3.9-year incidence of the first occurrence of the composite endpoint of an acute coronary syndrome, coronary revascularization, or stroke [15]. Furthermore, results from the TNT trial demonstrated that escalating MIS to HiS in T2D individuals with established coronary heart disease yields an incremental reduction of 25% in the incidence of major CV events [17]. In addition, the combination of statins to other classes of LLT, including Ez and PCSK9i, has been pursued as a compelling way to mitigate residual CV risk [16].

Notwithstanding current evidence supporting the reduction of CV outcomes with LLT, only 45.5% and 5.9% of individuals were, respectively, using statins and high-intensity LLT in our study. Similar results have been previously reported. Chamberlain et al. [38] demonstrated in a cross-sectional analysis of the Rochester Epidemiology Project that among individuals with T2DM and CVD, only 56.7% were on any statin regimen, of which 16.8% were on HiS and 42.1% had LDL-C <70 mg/dL [38]. Likewise, data from the Stable Coronary Artery Diseases Registry (START) found that prescription rates of combined therapy of statins with Ez and achievement of stricter LDL-C target (<55 mg/dL) in VHR subjects were, respectively, 4.8% and 3.2%, in consonance with our findings [39]. Despite consistent detachment from clinical guidance, treatment intensification of noncompliers also remains suboptimal. In that respect, Virani et al. [40] demonstrated that roughly one third of VHR individuals with uncontrolled LDL-C had their LLT intensified in the Veteran Affairs Cohort [40].

Reduction of SBP has also been pursued as a tempting target of CV prevention. In this matter, hypertension affects up to two thirds of T2D subjects, and data from observational studies support that hypertensive T2D individuals display a 1.6-fold higher CV mortality than their normotensive counterparts [41]. As each reduction of 10 mm Hg in SBP yields a 12% decrease in the incidence of T2D-related death and an 11% decrease in the incidence of myocardial infarction, stricter SBP reductions have emerged as a tempting target to lessen morbimortality [11].

In contrast with current evidence, our results and prior studies have found suboptimal SBP control in T2D [19,42]. In this regard, Khunti et al. [19] showed in a metanalysis with 24 studies including 369,251 people from 20 countries that only 29% of T2D individuals had SBP <130 mm Hg, while our study reported a 28.1% prevalence for the same target. Despite low attainment of the SBP target, optimal treatment with at least three classes of antihypertensive drugs was uncommon in our sample. In that respect, Fang et al. [42] demonstrated in the NANHES registry that three quarters of people with diabetes with SBP >140 mm Hg were using less than triple antihypertensive therapy and were thus eligible for antihypertensive therapy escalation with a significant impact on guidance compliance [42]. Comparably, in our study, among AHT-naïve individuals, prescription of triple AHT, which in Brazil is available for free in the public health system, would suffice to increase the control rate significantly.

Glycemic control has also been deemed to play a pivotal role in preventing CV events in T2D. Such a premise is grounded in many large randomized clinical trials and metanalysis showing that each 1% increase in A1c levels yields a 17% and 15% increase in major acute CV events and all-cause mortality, respectively [43,44,45]. Nevertheless, in a recent cross-sectional survey with 7760 individuals with diabetes from eight European countries, 53.6% of participants had HbA1c <7% [46]. In a more critical scenario, our study found that only 37.1% of individuals had HbA1c <7%, decreasing as estimated CV risk augmented.

In recent years, clinical management of individuals with T2D has moved from a long-lasting glucocentric perspective as recent clinical trials showed that new ADT classes improve CV outcomes irrespective of A1c reduction [47,48,49]. Despite accumulating evidence supporting these medications in T2DM patients at VHR, the prescription rate remains suboptimal. In that respect, Funck et al. [50] recently found in a prospective Danish cohort of VHR T2D subjects that only 14.7% of participants were on SGLT2i or GLP1a. Similarly, in our study, 16.6% of participants used cardioprotective ADT drugs; hence, the prescription of SGLT2i and GLP1a comprised 15.2% and 1%, respectively. It is worth noting that the prescription rate of these classes was comparable across CV risk categories, suggesting that estimated baseline CV risk played no influence on prescription.

### Study Limitations

Our study has limitations. Most importantly, adherence to therapy was not assessed in our study. This limited the accuracy of our results, as even individuals who were adequately prescribed may not have reached their respective targets due to low adherence. Furthermore, the Brazilian Diabetes Study was held in Campinas, SP, which presents a higher human development index and facilitated access to healthcare facilities than other Brazilian regions. When extrapolating our results to other middle-income regions, this should be borne in mind. Finally, as participation in this study demanded absence from work and may have been time-consuming, those who sought to participate in this clinical study were plausibly also more motivated and concerned about their health, which conceivably translated into higher rates of risk factor control.

## 5. Conclusions

A minority of T2D individuals comply with guideline recommendations for preventing CV events. This gap between current guidance and clinical practice is shortened by treatment intensification, and substantially impacts CV mortality.

## Figures and Tables

**Table 1:** Baseline characteristics per cardiovascular risk group.

	Overall	MR	HR	VHR	*p*-Value
*n*	1030	314	155	561	
Age. years	57.8 ± 8	54 ± 8.7	58 ± 8.4	59 ± 6.8	*0.001 ^a,b^*
Male. %	59.3	58.9	58.1	59.9	*0.905*
Married. %	72.2	72.9	73.5	71.5	*0.936*
Schooling. years	11 ± 4.2	11 ± 4.2	11 ± 3.7	10 ± 4.3	*0.006 ^c,b^*
Family income, USD	640 (760)	800 (800)	800 (600)	600 (740)	*0.001*
Caucasian, %	68.7	67.2	72.3	68.6	*0.510*
T2D duration, years	9.7 ± 7.3	4.7 ± 3.3	14 ± 4.5	10 ± 7.9	*0.001 ^a,b,c^*
Hypertension. %	81.4	61.1	73.5	94.8	*0.001*
Dyslipidemia. %	74.2	68.5	67.1	79.3	*0.001*
Prior CVD, %	17.4	0	0	31.9	*0.001*
Smoker. %	6.7	3.5	1.9	9.8	*0.001*
Obese. %	45.4	25.6	14.1	64.8	*0.001*
SBP, mm Hg	141 ± 20.4	135 ± 19.1	139 ± 19.4	144 ± 20.6	*0.001 ^b,c^*
DBP, mm Hg	83 ± 11.7	83 ± 10.3	83 ± 10.1	84 ± 12.7	*0.284*
**Biochemical analysis**					
Hemoglobin. mg/dL	14 ± 1.6	14 ± 1.6	14 ± 1.4	14 ± 1.6	*0.608*
HbA1c. %	7.9 ± 1.9	7.5 ± 1.8	8.1 ± 1.7	8.1 ± 1.9	*0.001*
Total cholesterol. mg/dL	182 ± 47.6	185 ± 51	181 ± 44	181 ± 47	*0.610*
LDL-C., mg/dL	107 ± 37.6	110 ± 37	109 ± 39	105 ± 38	*0.344*
HDL-C, mg/dL	44 ± 14.6	47 ± 19.5	46 ± 12.2	42 ± 11.9	*0.004*
VLDL-C, mg/dL	27 (15)	25 (14)	25 (14)	27 (16)	*0.030*
Triglycerides, mg/dL	159 (128)	145 (110)	148 (142)	164 (116)	*0.001*
Gfr, ml/min/1.73 m^2^	86 ± 18.1	91 ± 16	89 ± 15	82 ± 18	*0.010 ^a,c^*
Proteinuria. %	19.7	0	0	15.3	*0.001*
**Medications**					
Antihypertensive, %	64.4	42	52.3	80.2	*0.001*
Lipid-Lowering Therapy	45	35	41.9	51.3	*0.004*
*MiS*	36.3	29	34.8	40.8	
*HiS*	5.9	4.1	5.2	7.1	
*MiS-Ez*	1.3	0.6	1.3	1.6	
*HiS-Ez*	1.0	0.6	0	1.4	
*Ezetimibe*	0.5	0.6	0.6	0.4	
Antidiabetic therapy	98.5	97.1	99.4	99.1	*0.043*
**Control rate**					*<0.001*
HbA1c. %	41.2	51.1	33.6	38.4	*0.003*
SBP, %	30.6	23	26.4	16.1	*0.007*
LDL-C, %	18.8	42.8	14.4	7.8	*0.001*
Any two	16.2	27.4	7.0	13.1	*0.040*
All three	3.2	8.5	3.5	0.6	*0.001*

Pairwise comparison with *p* < 0.05 for *^a^* MR vs. HR, *^b^* MR vs. VHR and *^c^* HR vs. VHR. Data are presented as mean ± SD or median (IQR) for continuous variables and *n* (%) for categorical variables. MR, moderate risk; HR, high risk; VHR, very high risk; T2D, type 2 diabetes; CVD, cardiovascular disease; SBP, systolic blood pressure; DBP, diastolic blood pressure; MiS, moderate-intensity statin; HiS, high-intensity statin; MIS-Ez, MiS combined with ezetimibe; HiS-Ez, HiS combined with ezetimibe; HbA1c, glycated hemoglobin.

## Data Availability

The Brazilian Diabetes Study investigators will hold intellectual property over data. Its availability may be considered upon reasonable request.

## Supplementary Materials

The following supporting information can be downloaded at: https://www.mdpi.com/article/10.3390/diagnostics12040814/s1. Table S1. Supplementary baseline, Table S2: Logistic regression assessing the predictors of risk factor control attainment.

## Abbreviations

CVE, cardiovascular events; T2D, type 2 diabetes; SBP, systolic blood pressure; LDL-C, LDL cholesterol; A1c, glycated hemoglobin; CV, cardiovascular; CVD, cardiovascular disease; LLT, lipid-lowering treatment; CVR, cardiovascular risk; VHR, very high risk; HR, high risk; MR, moderate risk; eGFR, estimated glomerular filtration rate; ESC, European Society of Cardiology; EAS, European Atherosclerosis Society; MIS, moderate-intensity statin; HiS, high-intensity statin; Ez, ezetimibe; AHT, antihypertensive therapy; ADT, antidiabetic therapy.
