# Supplementary material for: Compliance with Cardiovascular Prevention Guidelines in Type 2 Diabetes Individuals in a Middle-Income Region: A Cross-Sectional Analysis
Source: Diagnostics (Basel). 2022 Mar 26;12(4):814. doi: 10.3390/diagnostics12040814 (PMC9024646; doi:10.3390/diagnostics12040814)
Supplement: Supplementary file 1 [file diagnostics-12-00814-s001.zip › diagnostics-1594923-supplementary.pdf]

Supplementary Material

Compliance with Cardiovascular Prevention Guidelines in Type 2 Diabetes Individuals in a Middle-Income Region: A Cross-Sectional Analysis

Joaquim Barreto [1], Beatriz Luchiari [1], Vaneza L. W. Wolf [1], Isabella Bonilha [1], Ticiane G. Bovi [1], Barbara S. Assato [1], Ikaro Breder [1], Sheila T. Kimura-Medorima [1], Daniel B. Munhoz [1], Thiago Quinaglia [1], Otavio R. Coelho-Filho [2], Luiz Sergio F. Carvalho [3], Wilson Nadruz [2] and Andrei C. Sposito [1,2,*]

[1] Atherosclerosis and Vascular Biology Laboratory (Aterolab), Division of Cardiology, State University of Campinas (Unicamp), Sao Paulo 13083-894, Brazil; joaquimbarretoantunes@gmail.com (J.B.); beatriz.m.luchiari@gmail.com (B.L.); vanessawolf@hotmail.com (V.L.W.W.); isaoliveira.ib@gmail.com (I.B.); ticianebovi@hotmail.com (T.G.B.); barbarasanae@gmail.com (B.S.A.); ikarobreder@gmail.com (I.B.); sheilatk@gmail.com (S.T.K.-M.); dbmunhoz@gmail.com (D.B.M.); tquinaglia@yahoo.com.br (T.Q.)
[2] Division of Cardiology, Department of Internal Medicine, Unicamp, Sao Paulo 13083-970, Brazil; tavicocoelho@gmail.com (O.R.C.-F.); wilnj@unicamp.br (W.N.)
[3] Clarity Healthcare Intelligence, Jundiaí 13214-658, Brazil; luizsergiofc@gmail.com
* Correspondence: sposito@unicamp.br; Tel.: +55-19-3521-7959

Table S1. Supplementary baseline characteristics

	Overall	MR	HR	VHR	*p-value*
n	1030	314	155	561	
**Demographics**					
Level of instruction. %					*0.004*
No formal education	29.4	27.7	19.4	33.1	
Primary school	36.1	32.2	45.2	35.8	
Secondary education	28.8	32.5	29	26.7	
University degree	5.7	7.6	6.5	4.5	
Ethnicity. %					*0.510*
Caucasian	68.7	67.2	72.3	68.6	
Black	11.2	11.1	8.4	11.9	
Asian	1.8	1.6	3.9	1.4	
Other	17.1	18.8	14.2	16.9	
**Medical history**					
T2D duration >10 years, %	45	2.9	100	50.6	*0.001*
CVD, %	17.4	0	0	31.9	*0.001*
Coronary heart disease. %	15	0	0	27.6	*0.001*
Previous ACS. %	9.1	0	0	16.8	*0.001*
CCS %	4.3	0	0	7.8	*0.001*
Previous PCI or CAGB. %	7.4	0	0	13.5	*0.001*
Stroke	2	0	0	3.7	*0.001*
Peripheral artery disease. %	1.1	0	0	2	*0.001*
Retinopathy. %	21	0	0	34.6	*0.001*
SBP Group					*0.001*
>140mmHg	48.5	25,3	42,9	57	
130-140mmhG	23.4	37	27,8	20,3	
<130mmHg	28.1	37,7	29,3	22,7	

LDL-C categories						*0.248*
	≥100 mg/dL	54.4	57.2	57.7	53.1	
	70-100mg/dL	30	28.9	27.9	30.1	
	55-70mg/dL	9.8.	9.8	12.3	9.0	
	<55mg/dL	5.9	4.1	2.1	7.8	
HbA1c range. %						*0.001*
	≥8%	41.2	31.4	48.6	44.5	
	7 to 8%	21.7	18.4	23.4	23	
	<7%	37.1	50.2	28	32.5	
**Medications**						
Antihypertensive, %		64.4	42	52.3	80.2	*0.001*
	ARB. %	45	30.3	37.4	55.3	*0.001*
	Beta-blocker. %	24.8	11.5	12.3	35.7	*0.001*
	Thiazide diuretics. %	24.2	15.6	18.7	30.5	*0.001*
	Calcium channel blocker. %	15.1	9.6	14.8	18.4	*0.001*
	ACEi. %	12.6	7.3	7.1	17.1	*0.001*
	Espironolactone. %	6.6	1.6	3.2	10.3	*0.001*
	Loop diuretics. %	5.5	1.9	1.9	8.6	*0.001*
	Hydralazine. %	0.6	0.3	0	0.9	*0.331*
	ACE or ARB, %	55.4	36	43.9	69.5	*0.001* [b,c]
Number of AHT classes, %						*0.001* [b]
	0	35,6	58	47,7	19,8	
	1	25	19,1	25,2	28,2	
	2	18,9	13.4	14.8	23.2	
	≥3	20,5	9.5	12.3	28.9	
Fibrates. %		6.1	5.1	5.8	6.8	*0.279*
AAS. %		24.4	8.6	16.8	35.3	*0.001*
P2Y12 inhibitors. %		3	0.6	0.6	5	*0.002*
Antidiabetic drugs						
	Metformin. %	98.5	97.1	99.4	99.1	*0.043*
	Sulphonylurea. %	40.8	33.1	52.9	41.7	*0.001*
	Dpp4i. %	18.7	19.1	29.7	15.5	*0.001*
	Sglt2i. %	15.2	15.6	20	13.7	*0.154*
	Pioglitazone. %	2.9	3.2	5.2	2.1	*0.133*
	GLP1a. %	1	0.3	1.3	1.2	*0.368*
	Insulin. %	19.5	13.7	20	22.6	*0.006*
Number of ADT						*0.001* [a]
	0	1.5	2.9	0.6	0.9	
	1	42.9	46.5	26.5	30.8	
	2	37.9	34.1	47.7	37.3	
	≥*3*	17.8	16.6	25.2	16.4	

T2D, type 2 diabetes; SBP, systolic blood pressure; LDL-C, LDL cholesterol; A1c, glycated hemoglobin; CV, cardiovascular; CVD, cardiovascular disease; LLT, lipid-lowering treatment; CVR, cardiovascular risk; VHR, very-high risk; HR, high-risk; MR, moderate-risk; eGFR, estimated glomerular filtration rate; MiS: moderate intensity statin; HiS, high-intensity statin; Ez, ezetimibe; AHT, antihypertensive therapy; ADT, antidiabetic therapy.

Table S2: Logistic regression assessing the predictors of risk factor control attainment

	**Univariate**				**Adjusted**			
**A1c**	OR (95%CI)	LB	UB	p value	OR (95%CI)	LB	UB	*p value*
Age	1.05	1.04	1.08	**< 0.001**	1.079	1.046	1.112	***< 0.001***
Male	0.95	0.71	1.27	0.740	0.824	0.538	1.262	*0.373*
Diabetes duration	0.95	0.92	0.97	**< 0.001**	0.947	0.915	0.979	***0.001***
Schooling	1.01	0.97	1.04	0.777	1.058	0.998	1.122	*0.060*
High income	1.28	0.89	1.83	0.180	1.477	0.930	2.346	*0.098*
BMI	0.99	0.96	1.02	0.368	0.991	0.945	1.038	*0.691*
CVR				**0.002**				*0.990*
MR	Ref				ref			
HR	0.59	0.43	0.83	**0.002**	0.535	0.264	1.083	*0.082*
VHR	0.49	0.30	0.78	**0.003**	0.578	0.336	0.993	***0.047***
**SBP**								
Age	0.96	0.95	0.98	**< 0.001**	0.973	0.946	0.999	***0.045***
Male	0.96	0.72	1.28	0.782	0.820	0.546	1.232	*0.340*
Diabetes duration	0.98	0.96	1.01	0.296	1.024	0.993	1.057	*0.130*
Schooling	1.04	1.01	1.09	**0.007**	1.056	0.997	1.118	*0.065*
High income	1.63	1.13	2.36	**0.010**	1.307	0.843	2.026	*0.231*
BMI	0.99	0.96	1.02	0.424	1.003	0.960	1.048	*0.894*
CVR				**<0.001**				***0.018***
MR	Ref							
HR	0.72	0.47	1.11	0.138	0.606	0.319	1.151	*0.126*
VHR	0.45	0.33	0.63	**< 0.001**	0.473	0.282	0.794	***0.005***
**LDL-C**								
Age	0.98	0.95	1.00	0.053	1.021	0.984	1.060	*0.262*
Male	1.53	1.01	2.32	**0.047**	1.678	0.937	3.006	*0.082*
Diabetes duration	0.95	0.92	0.99	**0.004**	1.020	0.975	1.067	*0.380*
Schooling	1.04	0.99	1.09	0.076	0.992	0.916	1.073	*0.833*
High income	1.39	0.88	2.21	0.157	1.222	0.657	2.227	*0.527*
BMI	0.96	0.92	0.99	**0.043**	1.033	0.970	1.099	*0.313*
CVR				**< 0.001**				***< 0.001***
MR	Ref				Ref			
HR	0.22	0.12	0.43	**< 0.001**	0.170	0.069	0.417	***< 0.001***
VHR	0.11	0.07	0.18	**< 0.001**	0.084	0.039	0.181	***< 0.001***
**Any two**								
Age	1.01	0.98	1.03	0.929	1.037	0.998	1.078	*0.063*
Male	0.88	0.59	1.34	0.560	0.730	0.415	1.284	*0.274*
Diabetes duration	0.94	0.91	0.98	**0.001**	0.979	0.933	1.027	*0.385*
Schooling	1.05	0.99	1.10	0.088	1.131	1.044	1.225	***0.003***
High income	1.46	0.89	2.39	0.134	1.090	0.582	2.041	*0.787*
BMI	0.99	0.96	1.04	0.876	1.038	0.976	1.104	*0.236*
CVR				**< 0.001**				***< 0.001***
MR	Ref				Ref			
HR	0.21	0.09	0.45	**< 0.001**	0.212	0.078	0.578	***0.002***
VHR	0.28	0.18	0.44	**< 0.001**	0.196	0.095	0.401	***< 0.001***
**All three**								

Age	0.98	0.93	1.05	0.697	1.042	0.956	1.135	*0.352*
Male	1.20	0.47	3.09	0.703	1.010	0.260	3.922	*0.988*
Diabetes duration	0.92	0.84	1.01	0.064	0.965	0.831	1.120	*0.640*
Schooling	1.06	0.94	1.19	0.366	0.995	0.827	1.196	*0.954*
High income	1.47	0.41	5.31	0.549	1.262	0.264	6.031	*0.771*
BMI	0.88	0.79	0.99	**0.041**	0.969	0.822	1.142	*0.706*
CVR				**< 0.001**				*0.126*
MR	Ref				Ref			
HR	0.39	0.11	1.38	0.145	0.240	0.019	3.040	***0.271***
VHR	0.06	0.01	0.28	**< 0.001**	0.146	0.022	0.974	***0.047***

Logistic regression using attainment to A1c, SBP, LDL-C, any two and all three goals as dependent variables. OR, odds ratio; CI, confidence interval; BMI, body mass index; CVR, cardiovascular risk; MR, moderate risk; HR, high risk; VHR, very-high risk
